# Perichondrial progenitor cells promote proliferation and chondrogenesis of mature chondrocytes

**DOI:** 10.1093/rb/rbab078

**Published:** 2022-01-04

**Authors:** Chien-Liang Ho, Lynn L H Huang, Shyh-Jou Shieh

**Affiliations:** Institute of Clinical Medicine, College of Medicine, National Cheng Kung University, 138 Sheng-Li Road, Tainan 70403, Taiwan; Division of Plastic and Reconstructive Surgery, Department of Surgery, National Cheng Kung University Hospital, College of Medicine, National Cheng Kung University, 138 Sheng-Li Road, Tainan 70403, Taiwan; Research Center of Excellence in Regenerative Medicine, National Cheng Kung University, 1 University Road, Tainan 70101, Taiwan; Division of Plastic and Reconstructive Surgery, Department of Surgery, National Cheng Kung University Hospital, College of Medicine, National Cheng Kung University, 138 Sheng-Li Road, Tainan 70403, Taiwan; Research Center of Excellence in Regenerative Medicine, National Cheng Kung University, 1 University Road, Tainan 70101, Taiwan; Department of Biotechnology and Bioindustry Sciences, College of Bioscience and Biotechnology, National Cheng Kung University, 1 University Road, Tainan 70101, Taiwan; International Center for Wound Repair and Regeneration, National Cheng Kung University, 1 University Road, Tainan 70101, Taiwan; Division of Plastic and Reconstructive Surgery, Department of Surgery, National Cheng Kung University Hospital, College of Medicine, National Cheng Kung University, 138 Sheng-Li Road, Tainan 70403, Taiwan; International Center for Wound Repair and Regeneration, National Cheng Kung University, 1 University Road, Tainan 70101, Taiwan; School of Medicine, National Cheng Kung University, 1 University Road, Tainan 70101, Taiwan

**Keywords:** chondrocytes, cell proliferation, perichondrial progenitor cells, paracrine effect, neocartilage formation

## Abstract

Autologous chondrocytes (C cells) are effective sources of cell therapy for engineering cartilage tissue to repair chondral defects, such as degenerative arthritis. The expansion of cells with C cell characteristics has become a major challenge due to inadequate donor sites and poor proliferation of mature C cells. The perichondrial progenitor cells (P cells) from the cambium layer of the perichondrium possessed significantly higher mesenchymal stem cell markers than C cells. In the transwell co-culture system, P cells increased the passaging capacity of C cells from P6 to P9, and the cell number increased 128 times. This system increased the percentage of Alcian blue-positive C cells from 40% in P6 to 62% in P9, contributing about 198 times more Alcian blue-positive C cells than the control group. C cells co-cultured with P cells also exhibited higher proliferation than C cells cultured with P cell-conditioned medium. Similar results were obtained in nude mice that were subcutaneously implanted with C cells, P cells or a mixture of the two cell types, in which the presence of both cells enhanced neocartilage formation *in vivo*. In aggregate, P cells enhanced the proliferation of C cells in a dose–dependent manner and prolonged the longevity of mature C cells for clinical applications.

## Introduction

Cartilage damage possesses poor self-repair capability, which is correlated with size, depth, position of the defect and age of patients [1]. When left untreated, cartilage defects continue to worsen over time and may result in one of the morbidities known as arthritis. As a consequence, an effective method for treating cartilage defects or improving the poor intrinsic regenerative capacity of cartilage is urgently needed [2, 3].

Clinicians have used cell therapy, tissue engineering or artificial implantation to heal cartilage defects [4, 5]. Although implant replacement is currently a popular and pragmatic solution, it remains limited, complicated and expensive. According to some clinical reports, autologous chondrocyte (C cell) transplantation provides a direction for treating cartilage defects of limited size with an acceptable outcome [6]. Large cartilage defects increase the difficulty of harvesting a high number of autologous C cells with donor site morbidity. The concept of tissue engineering involves the utilization of a combination of potential cells, an optimal scaffold, and specific factors to build sufficient tissue *in vitro* for cartilage regeneration. Tissue engineering products can be implanted into any cartilage defect without the accompanying complications of artificial implants. Various cell types have been used, such as mesenchymal stem cells (MSCs), adipose-derived stem cells and adult progenitor cells, under different culture conditions [5, 7–9].

The three main disadvantages of autologous C cells for cartilage repair and reconstruction are: (i) fewer C cell donor sites; (ii) de-differentiation of C cells after cell culture expansion; and (iii) early apoptosis of C cells [4, 10–12]. To overcome these drawbacks, it is essential to find alternative cartilage repair cells for clinical practice. The criteria for such cells would include a higher rate of proliferation, self-renewal capability and cooperative interaction with mature C cells by secreting factors that enhance or maintain the chondrogenic phenotype.

Various cell types, such as MSCs, adipose-derived stem cells and various types of progenitor cells, have been explored and demonstrated potential for cartilage regeneration [2, 3, 13]. MSCs are prone to differentiate into various cell lineages, and specific induction manipulation is required for chondrogenesis; however, the percentage of C cells is variable. Some unipotent progenitor cells for cartilage formation have been found in special tissue types, such as the perichondrium, synovial membrane and fascia [14, 15].

The perichondrium can promote cartilage repair, and progenitor cells possess high potential for cell proliferation while maintaining their original phenotype [16–18]. Harvesting P cells from auricular cartilage offers at least two advantages: (i) auricular cartilage is easy to harvest; and (ii) it has minimal donor-site morbidity [19]. Autologous C cells are a reliable cell source for cartilage regeneration [20]. Most literature [12, 21–23] has focused on regeneration and differentiation of bone and tympanic membranes, and no reports currently exist regarding the interaction between P cells and mature C cells. This study isolated progenitor cells and mature C cells from the perichondrium and cartilage of rabbit ears to elucidate their interaction, proliferation and chondrogenesis. We found that progenitor cells can improve the shortage of autologous C cells, and possess great potential for cartilage regeneration in future clinical applications.

## Materials and methods

### Cell harvest and culture

**Figure 1.**
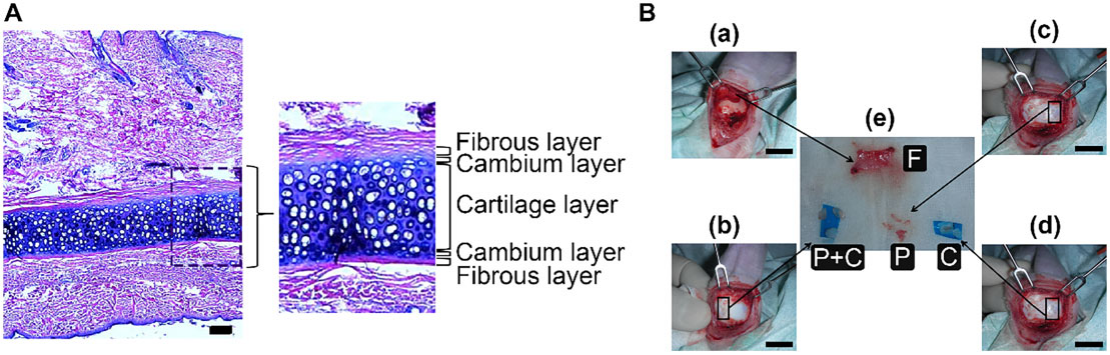
Auricular cartilage dissection and tissue separation. (**A**) The tissue of the anatomical structure of a rabbit ear stained with hematoxylin and eosin, scale bar: 100 Tm. (**B**) A schematic illustration of different tissues harvested from an ear of a 4-weeks-old New Zealand white rabbit, scale bar: 1 cm (a) a dense fibrous layer of perichondrium was elevated, and F cells were harvested. (b) Beneath the fibrous layer, the superior layer of cartilage was peeled to obtain P cells and C cells (P + C cells). (c) The opposite side of cartilage was scraped to obtain P cells. (d) After scraping, the tissue underneath was peeled to obtain C cells. (e) Collection of various tissues for harvesting different cell types

The cells were isolated from male 4-weeks-old New Zealand white rabbits purchased from the Taiwan Livestock Research Institute, Council of Agriculture, Executive Yuan. The rabbits were raised at the animal center of the National Cheng Kung University (NCKU), following the IACUC No. 97177 and 100028 protocols approved by the NCKU Animal Research Committee. The rabbits were anesthetized intraperitoneally with Zoletil 50 (tiletamine/zolazepam, Virbac, France) and 2% Rompun (xylazine hydrochloride, Bayer, France). During surgery, the rabbits were continuously anesthetized using a subcutaneous circumferential injection of 2% xylocaine (AstraZeneca AB, Sweden) into the root of the ear. Then, 95% alcohol was used to sterilize the surface of the ear, which was covered with a sterilized cloth. The anatomy of the New Zealand white rabbit ear is shown in Fig. 1A.

To harvest various cell types, a No. 15 blade was used to make a longitudinal and a horizontal incision to create a piece of skin–muscle flap in the ear root, which was elevated to expose the fibrous layer, perichondrium and cartilage, as described in Fig. 1B. A periosteal elevator was used to separate the fibrous layer from the perichondrium. The fibrous layer was harvested from the connective tissue between the muscle and perichondrial areas to obtain fibroblasts (F cells). Subsequently, another No. 15 blade was used to peel the perichondrium together with mature cartilage to harvest P cells and mature C cells (P +  (P +ocytemature enitoras used to separate the fibrous layer from the perichondrium. The fibrous layer waP cells. After the perichondrium was harvested, the scraped area was peeled with a new blade to obtain C cells (Fig. 1B).

The F, P, C and P + C cells were harvested from tissues in the proximal portion of the rabbit ear root. The tissues were washed in phosphate-buffered saline (PBS) without magnesium and calcium ions (pH 7.4, Sigma-Aldrich, USA) and digested with 1 mg/ml collagenase (100 U/mg) (Worthington Biochemical, USA) with gentle shaking (60 rpm) at 37°C for at least 1–4 h. The digests were passed through a 100-μm nylon mesh, and the cells were harvested. The cells in Dulbecco’s modified Eagle’s medium-low glucose (DMEM-LG; Gibco, USA) with 10% fetal bovine serum (FBS; Gibco, USA) and gentamicin (0.05 mg/ml; Sigma-Aldrich, USA) were seeded at a density of 2000 cells/cm^2^ in 6-well plates (Greiner Bio-One North America, USA) and incubated at 37°C with 5% CO_2_.

### Flow cytometry

The cells were harvested by trypsinization, washed and suspended in PBS. To analyze the characteristics of MSCs, the cells were incubated with three antibodies of surface-specific markers CD44, CD90 and CD105 at 4°C for 30 min in the dark, and then characterized using flow cytometry (FACS Calibur; BD Biosciences, USA). CD44 antibody was conjugated with phycoerythrin (Abcam, USA), CD90 antibody with fluorescein isothiocyanate (Abcam, USA) and CD105 antibody with allophycocyanin (Abcam, USA). The expression percentage of cells was analyzed using a Windows Multiple Document Interface software (WinMDI 2.0; The Scripps Research Institute, USA).

### Transwell co-culture system

P cells were cultured at different cell densities (1000, 2000, 3000 and 4000/cm^2^) in transwell inserts with a 0.4-μm-pore membrane, which separated P cells in the upper chamber from 2000 cells/cm^2^ of C cells in the lower chamber (Fig. 3A). The cells were cultured in DMEM-LG and 10% FBS, and the medium was changed every 72 h until the cells reached subconfluence (90% ± 5%). The control was the C2000 group, while the experimental groups had various numbers of P cells, i.e. P4000/C2000, P3000/C2000, P2000/C2000 and P1000/C2000, respectively, in the above transwell co-culture system (Fig. 3A). Cumulative population doubling was calculated using the equation below.

### Conditioned medium system

In the conditioned medium system, C cells (2000 cells/cm^2^; C2000) were cultured in 24-well plate with conditioned media obtained from variable densities of P cells (1000, 2000, 3000 and 4000/cm^2^) in 12-well plates (Greiner Bio-One, Germany) and denoted as P1000→C2000, P2000→C2000, P3000→C2000 and P4000→C2000, respectively. The media were changed every 24 h, including the control group, C2000, without conditioned medium, as depicted in Fig. 4A. The cell passage criterion was the same when the C cells reached subconfluency, which was defined as 90% ± 5% of confluency. For the P4000→C2000^§^ group, either P cells or C cells passaged independently while reaching subconfluency, and the conditioned medium from P cells was used to culture C cells and changed every day. Cumulative population doubling was calculated using the equation below.

### Proliferative calculation of cumulative population doubling

To understand the potential of P cells for enhancing the proliferation of C cells, the cumulative cell numbers at various passages were calculated according to the cumulative population doubling. A total of 4000 cells were consistently seeded initially in each well of a 24-well plate for subcultures. At subconfluence, the cells were trypsinized and the numbers were counted.

The cumulative population doubling = log of (final cell number at subconfluence/initial cell number seeded)/log2.

The initial seeded cell number was 4000 and *C*_1_ was the first value, representing the cell number at subconfluence in the first passage, and the cumulative cell number for the *n*th passage, which was calculated based on the following formula:

$$C_{n}=\text{cell number at subconfluence of the}\;\mathrm{nth}\;\text{passage}\times C_{n-1}{}_{/}4000.$$

Here, C_*n*_ represents the final cumulative cell number.

### *In vitro* staining with Alcian blue

Glycosaminoglycans bear strong negative charges, which can be detected by Alcian blue staining. When the above C cells were subconfluent, they were fixed with 4% paraformaldehyde for 30 min, washed with PBS three times, and then stained with 1% Alcian blue (74 240; Chroma-Gesellschaft, Germany) in 0.1 N hydrochloric acid solution (pH 1.0) for 30 min. Data were scanned and analyzed using ImageJ software (ImageJ 1.47; National Institutes of Health, USA).

### Surgical implantations

Six-weeks-old male nude mice (*n *=* *17) were subcutaneously implanted with cell-embedded gels containing C, P, P + C or F cells (control group) harvested from rabbit ears (five mice per test group and two mice in the control group). Each cell-embedded gel contained 1 ×10^7^ cells with 450 cl of cell tissue gel, and the gel was comprised of collagen and hyaluronan prepared according to a patent [24]. The mice were maintained in accordance with the NCKU guidelines for the care and use of laboratory animals. All experimental protocols and surgical procedures were approved by the Institutional Animal Care and Use Committee under protocols IACUC No. 97177 and 100028. The mice were anesthetized with an intraperitoneal injection of ketamine. After sterilization, cell-embedded gels with various cells at ∼5000l were subcutaneously inserted into the back of each mouse after a straight-line incision was made in the center of the back. The incision was then closed using non-absorbable sutures. Mice in the control group had a cell-embedded gel mixed with F cells inserted into their backs.

### Histological examination

The mice were euthanized 8 weeks after the surgical implantation. The implants and surrounding tissues were removed from the inserted sites and fixed in 10% neutral-buffered formalin. Consecutive sections were cut from the paraffin blocks and fixed on 5-mm slides, deparaffinized, and stained with hematoxylin and eosin to assess the morphology of their neocartilage using an optical microscope (BX51; Olympus, Japan) and panoramic view was recorded using TissueGnostics FACS-like Tissue Cytometry (TissueFAXS Plus; TissueGnostics GmbH, Austria). Alcian blue staining combined with nuclear fast red staining (1A402; Chroma-Gesellschaft, Germany) was used to evaluate glycosaminoglycans and cell nuclei in neocartilage. The Alcian blue-positive areas were quantified using an ImageJ software.

### Statistical analysis

Student’s *t-*test was used to examine differences between the groups. Significance was set at *P < 0.05 and **P <* *0.01.

## Results

### Separation of perichondrium from cartilage

The proximal region of the New Zealand white rabbit ear was anatomically examined. The micrograph of the auricular tissue illustrates the relationship between the perichondrium and cartilage (Fig. 1A). The perichondrium is composed of two layers: the outer fibrous layer and the inner cambium layer. The outer fibrous layer contains F cells; whereas, the inner cambium layer contains small and flat P cells. In addition, the perichondrium is near the cartilage, which is composed of C cells that produce a large amount of extracellular matrix without vascular supply (Fig. 1A).

In order to mimic clinical practice for future applications, we used the surgical dissection method to separate different tissue layers for harvesting different cell types (P, C or F cells). After the dissection of skin and muscular layers from the proximal to the distal part of the rabbit ear, the fibrous layer was dissected and elevated using a periosteum elevator to expose the perichondrial layer [Fig. 1B(a)]. The F cells were obtained from the fibrous layer. In Fig. 1B(b), the perichondrium and the part of cartilage was peeled, and P + C cells were harvested. As shown in Fig. 1B(c), the perichondrium was scraped with surgical blade No.15 to obtain P cells next to the P + C cells harvested site. Underneath the perichondrium harvest site, cartilage was peeled using a surgical blade [Fig. 1B(d)], and pure C cells were obtained. Four different tissue groups in Fig. 1B(e) were dissected into small pieces and digested with collagenase to harvest the respective cells as described above.

### P cells express higher stem cell characteristics than C cells

**Figure 2.**
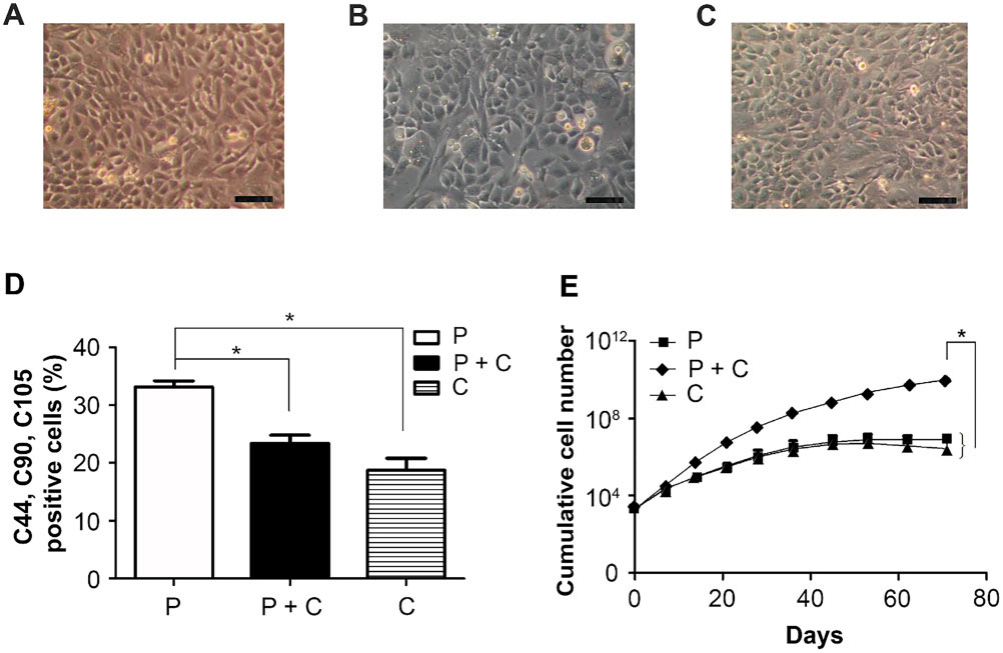
Characterization and cell growth of P + C cells. Optical micrographs of the (**A**) P cells, (**B**) P + C cells and (**C**) C cells. (**D**) Quantification of CD44, CD90 and CD105 triple-positive cells in the groups of P, P + C and C, respectively (*n *=* *3) (*P <* *0.05). (**E**) The cumulative cell number of three groups at different time points. Each group was passaged at the same time point (*n *=* *3) (*P <* *0.05)

The cell morphologies of the P cells, P + C cells and C cells are shown in Fig. 2A–C. To identify whether P, C, and P + C cells have the characteristics of MSCs, the expression of surface markers CD44, CD90 and CD105 was examined using flow cytometry. The data revealed that these cells were positive for CD44, CD90 and CD105, and the P cell group had a significantly higher ratio of CD44^+^/CD90^+^/CD105^+^ cells than the P + C and C cell groups (Fig. 2D). The P + C cell group showed a significantly higher proliferation rate, while the C cell group showed a slight decrease in proliferation rate after Day 60 (Fig. 2E). Based on the proliferation of P + C cells, the following experiments were conducted to study the paracrine effects of P cells on the proliferation of C cells.

### Co-culture with P cells promoted proliferation and characteristics of C cells

**Figure 3.**
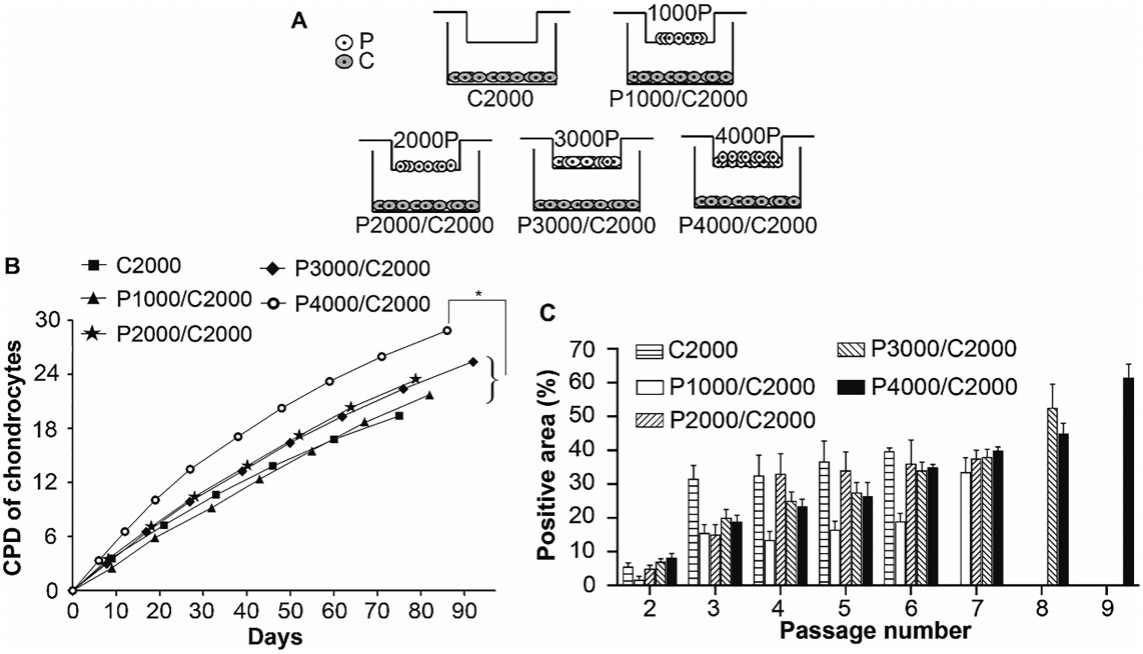
Effects of P cells in co-culture system on the proliferation of C cells. (**A**) The experimental scheme indicates abbreviated forms of 5 groups with P in the upper chamber co-cultured with C in the lower chamber in a transwell system. (**B**) The cumulative population doubling of C when co-cultured with different densities of P (*n *=* *3) (*P <* *0.05). (**C**) The percentage of Alcian blue-positive areas indicating the chondrogenic expression of the five groups cultured for nine passages. The missing data were due to cessation of cell growth

A co-culture system (Fig. 3A) was established to investigate the paracrine effects of P cells in transwell inserts on promoting C cell proliferation, and the cumulative population doubling of C cells in the lower chambers was calculated. The data demonstrated that C cells co-cultured with a higher number of P cells showed greater proliferation than C cells cultured alone (Fig. 3B). Since Alcian blue is typically used to evaluate the characteristics and functional expression of C cells, it was used to stain C cells in the lower chambers. The missing data after the sixth passage in Fig. 3C indicate that C cells lost their proliferative capacity to reach subconfluent criteria for passages. In contrast, C cells co-cultured with higher numbers of P cells could prolong the lifespan of C cells. The C cells in the P4000/C2000 group had the longest lifespan, and the percentage of Alcian blue staining positive area increased steadily along with the passage number; however, the cell numbers at subconfluency were similar. The same trend was observed in the P3000/C2000 and P2000/C2000 groups; however, the lifespans of C cells decreased gradually to the eighth and seventh passages, respectively. There was no significant difference in the maximum percentages of Alcian blue staining positive areas among the groups of C2000, P1000/C2000 and P2000/C2000. However, a higher number of P cells led to a higher maximum percentage of Alcian blue staining positive areas in C cells in the P3000/C2000 and P4000/C2000 groups. These data indicate that P cells have paracrine effects on prolonging the lifespan and characteristics of C cells.

### P cells increased expansion of C cells, and largely enhanced the characteristics of C cells among C cells

To clarify the expansion magnitude of C cells and the extent of those with characteristic chondrocytes promoted by P cells in a transwell co-culture system, it was assumed that each cell occupied the same area in each individual group. The cumulative cell numbers in Table 1 were calculated from each end passage of the total cell numbers in the respective transwell co-culture system. The expansion magnitude was calculated using the equation: (cumulative cell number)/(initial number of seeded cells), and the initial seeding cells were 4000 cells/well. C cells in the P4000/C2000 group were prolonged to more than the ninth passage (Fig. 3C); therefore, the expansion magnitude increased to 148.3 × 10^4^, and the ratio of cumulative cell number became 128 times that of the control C2000 group.

**Table 1.** Expansion of C cells affected by transwell co-culture system of variable numbers of P cells

Group	Passage number	Cumulative cell number^a^ (×10^7^)	Expansion magnitude^b^ (×10^4^)	Ratio of cumulative cell number^c^	Alcian blue^+^ cell number estimated^d^ (×10^7^)	Ratio of Alcian blue^+^ cells^e^	Alcian blue^+^ cells (%)^f^
C2000	P6	4.63 ± 0.01	1.2	1	1.84 ± 0.00	1	40
P1000/C2000	P7	13.10 ± 0.12	3.3	3	4.39 ± 0.09	2	34
P2000/C2000	P7	52.46 ± 0.72	13.1	11	19.67 ± 0.25	11	38
P3000/C2000	P8	104.8 ± 1.0	26.2	23	55.0 ± 1.9	30	53
P4000/C2000	P9	593.1 ± 7.4	148.3	128	364.8 ± 7.3	198	62

a Cumulative cell number: total number of cumulative cells in the indicated passage.

b Expansion magnitude was calculated using the equation of (cumulative cell number)/(initial number of seeded cells).

c Ratio of cumulative cell number = (cumulative cell number)/(cumulative cell number of the C2000 group).

d Alcian blue^+^ cell number: based on cell number of each group, the Alcian blue^+^ cell number was calculated according to the percentage of Alcian blue^+^ area.

e Ratio of Alcian blue^+^ cells = (Alcian blue^+^ cell number)/(Alcian blue^+^ cell number of the C2000 group).

f Alcian blue^+^ cells (%) = (Alcian blue^+^ cell number/cell number of the respective group) × 100.

The Alcian blue^+^ cell numbers were calculated from the percentages of Alcian blue^+^ area (Fig. 3C) based on the above assumption and the total cell number at subconfluence in each group. The ratio of Alcian blue^+^ cells was calculated from the equation: (Alcian blue^+^ cell number)/(Alcian blue^+^ cell number of the C2000 group), as shown in Table 1. Finally, the percentage of Alcian blue^+^ cells in each group was calculated using the following equation: (Alcian blue^+^ cell number)/(cell number of the respective group)×100%. Our data revealed that the number of Alcian blue^+^ cells steadily increased depending on the number of P cells co-cultured with C cells (Table 1). In particular, the Alcian blue^+^ cells were 2, 11, 30 and 198 times higher in the groups of P1000/C2000, P2000/C2000, P3000/C2000 and P4000/C2000, respectively, than in the control group of C2000 (Table 1). Overall, the percentage of Alcian blue^+^ cells increased from 40% to 62%, indicating that P cells promoted the C cell characteristics of C cells.

### P cells in transwell system stimulated the best proliferation of C cells

**Figure 4.**
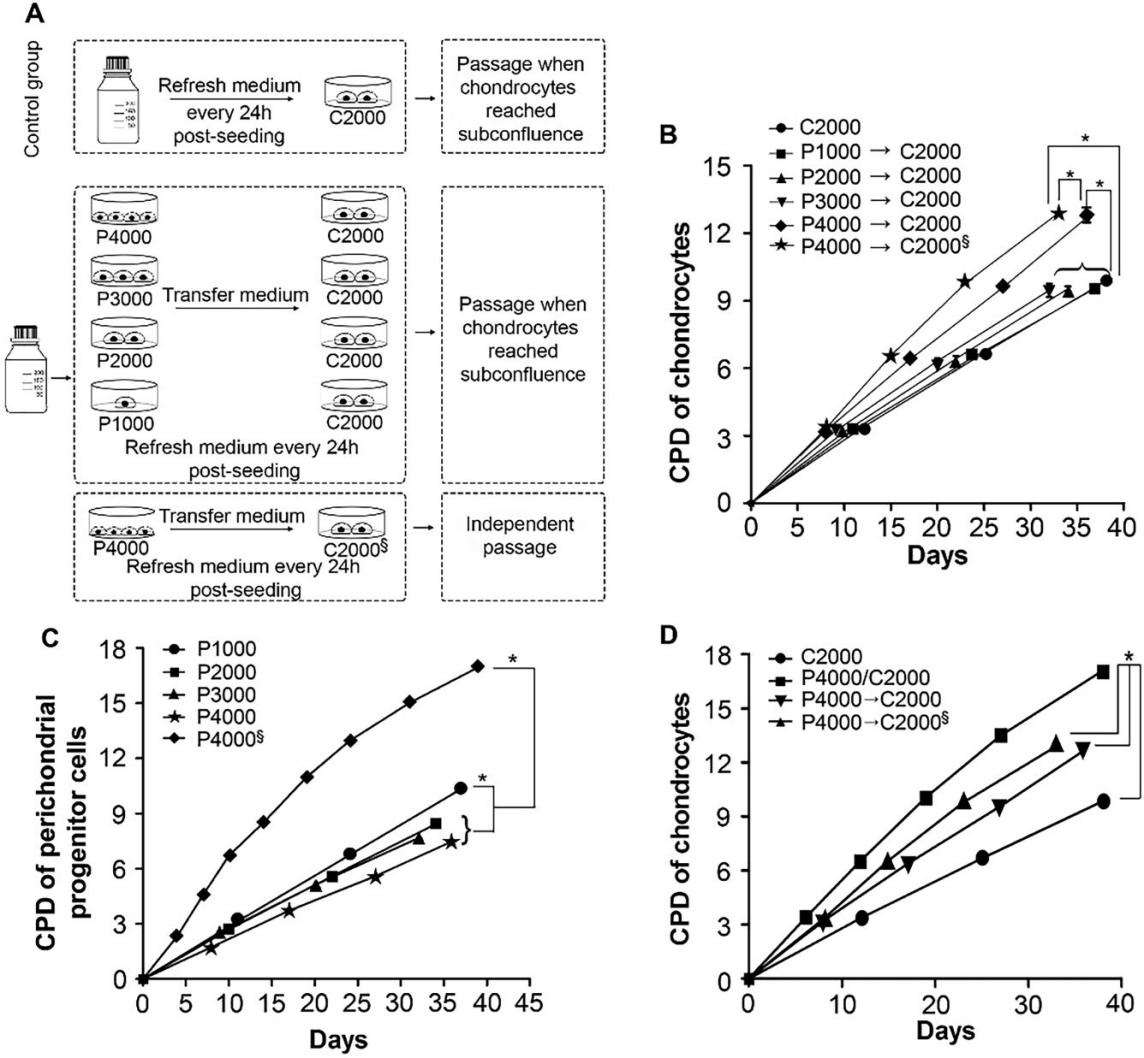
Effects of P cells in different culture systems on the proliferation of C cells. (**A**) The experimental design represents a control with C2000 only, conditioned medium from each group of P cells to feed C cells daily following the passage criteria of C2000 in the next four groups, and in the P4000/C2000^§^ group following individual passage of P cells and C cells. (**B**) The cumulative population doubling of C cells in the conditioned medium system at different time points (*n *=* *3) (*P <* *0.05). (**C**) The cumulative population doubling of P cells at different time points (*n *=* *3) (*P <* *0.05). (**D**) The comparison of the cumulative population doubling of C cells in different culture systems and passage criteria (*n *=* *3) (*P <* *0.05)

To further investigate the paracrine effects on P cells, various C cell groups were cultured in different conditioned media that were transferred from the cultures of different P cell numbers (Fig. 4A). When C cells were cultured in the conditioned media, which were transferred from a higher number of P cells, the cumulative population doublings of the C cells increased concomitantly (Fig. 4B). In addition, the P4000→C2000^§^ group, in which P + C cells were passaged-independently according to each other’s subconfluency criteria, had the highest cumulative population doubling of C cells among the six groups (*P <* *0.05). To determine the rationale for the discrepancy between the P4000→C2000 and P4000→C2000^§^ groups, the cumulative population doublings of P cells in the groups were analyzed. As shown in Fig. 4C, the P cells in groups P1000, P2000, P3000 and P4000 were passaged only when their corresponding C cells reached the required subconfluence; whereas, the P cells in group P4000^§^ were passaged when they reached subconfluence. Higher numbers of P cells led to lower cumulative population doublings in the P1000, P2000, P3000 and P4000 groups, which were passaged with their corresponding C cells at the required subconfluent criteria. In contrast, the P cells in the P4000^§^ group had the highest cumulative population doubling among the five groups (*P <* *0.05).

Moreover, the cumulative population doublings of C cells in various culture systems were compared in Fig. 4D. In the transwell co-culture system, the C cells in the P4000/C2000 group showed the highest cumulative population doubling, which may be due to factors secreted by the P cells into the culture medium. Although the C cells in both groups of P4000→C2000^§^ and P4000→C2000 were influenced by the conditioned media of P cells, the cumulative population doublings of C cells diminutively declined. Due to the absence of P cells in the culture condition, the lowest cumulative population doubling was found in the C2000 group.

### P cells promoted neocartilage formation *in vivo*

**Figure 5.**
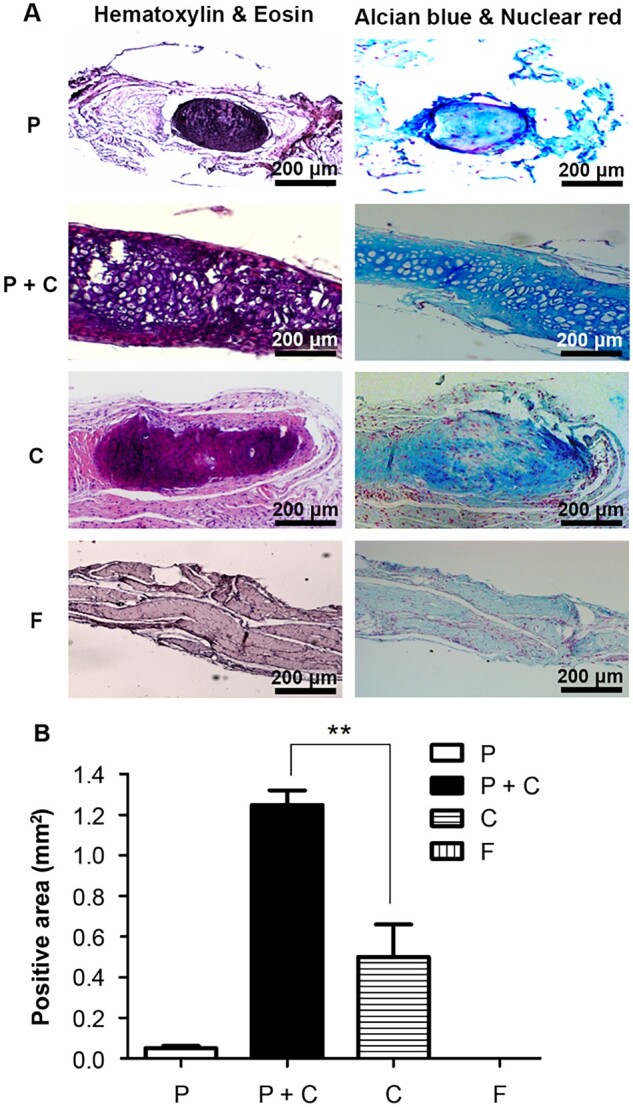
Histological examination of various cell types. (**A**) The four groups of cells mixed with cell tissue gel and implanted subcutaneously in the back of nude mice. The specimens were harvested 2 months later and examined histologically with Alcian blue and nuclear fast red staining. (**B**) The percentages of Alcian blue-positive areas representing the chondrogenic expression were quantified in the four groups as indicated (*n *=* *2) (**P <* *0.01)

To demonstrate the value of P + C cells and to diminish the environmental interference on chondrogenesis, the subcutaneous implantation model was chosen to elucidate the role of P cells in promoting cartilage formation. Various groups of cells were subcutaneously injected into the backs of nude mice, and histological examination was performed. In Fig. 5A, Alcian blue and nuclear red staining was used for the expression of glycosaminoglycans in the extracellular matrix of cartilaginous tissue and cell nuclei, respectively. The results in the P + C cell group demonstrated the greatest expression with the highest intensity of Alcian blue-positive area, which revealed significant neocartilage formation (Fig. 5A). It was also found that not only did P cells largely enhance the expansion of the C cells, but the interaction of both cells showed a synergetic effect on chondrogenesis. The group implanted with C or P cells also formed neocartilage tissues subcutaneously, although the group of P cells did not express a typical architecture of cartilage. No neocartilage formation was observed in the group implanted with the F cells. The quantitative data were presented in Fig. 5B, and the positive area of Alcian blue staining of the P + C cell group was 2.4 times larger than that in the group implanted with C cells.

## Discussion

Insufficient resources and the poor proliferative ability of C cells limit their application in clinical practice. This study provides an expansion method of C cells for cartilage regeneration and clinical applications. It was found that the paracrine effects of P cells in the transwell co-culture system dose-dependently promoted the proliferation and cartilaginous expression of C cells, and prolonged the lifespan of C cells (Fig. 3). It was also determined that the transwell co-culture system was superior to the conditioned medium system, in which the paracrine effects of P cells directly influenced C cells. As shown in Fig. 4D, the P4000/C2000 group had better proliferation potential than the P4000→C2000 and P4000→C2000^§^ groups. The results also showed that C cells co-cultured with P cells had better proliferation and neocartilage formation than C cells or P cells alone (Figs 2B and 5). The paracrine factors could be growth factors and chemokines secreted from P cells to modulate adjacent cellular responses.

Perichondrium is a dense connective tissue surrounding cartilage, and it contains various types of cells for cartilage and bone development [25]. In addition, it has been reported that the inner (cambium) layer of the perichondrium provides cells for neocartilage formation, and the outer (fibrous) layer rapidly produces a fibrous tissue overgrown to restore the mechanical function of the cartilage [14, 15, 26]. Cartilage progenitor cells have been identified in the perichondrium of the adult ear, particularly in the cambium layer [27, 28]. This study further confirmed that the inner cambium layer contained small and flat P cells (Fig. 1A).

In this study, the characteristics of the cells were investigated using MSC-specific markers. In a previous study, CD105, CD73 and CD90 were reported as criteria for MSCs, and Kobayashi *et al.* [28, 29] also noted higher expression of CD44 and CD90 in perichondrocytes compared to chondrocytes. Our data showed that the P cells expressed higher amounts of CD44, CD90 and CD105 triple-positive cells than did the C cells (Fig. 2D), indicating a higher potential of MSCs in the P cells.

In a separate experiment (data not shown), a tissue digestion method was used with collagenase treatment for 2 h to harvest P cells, and then further digested the rest of the cartilage to harvest C cells. A characteristic clonality of stem cells was observed in the P cells harvested by this method and the above surgical dissection method.

C cells co-cultured with P cells were significantly more proliferative than P cells or C cells cultured individually (Fig. 2E). A few studies have demonstrated that MSCs or adipose-derived stromal cells co-cultured with C cells promote C cell proliferation [30, 31]. Tsuchiya *et al.* [32] reported an elevated proliferation rate of bovine mature articular C cells when co-cultured with MSCs and upregulation of their differentiation into a chondral phenotype in a mixed pellet culture. These results indicate that mature C cells can be promoted by stem cells or progenitor cells.

Furthermore, this study found that P cells promoted C cell proliferation in a dose–dependent manner (Fig. 3B). Particularly, P cells co-cultured with C cells at a 2:1 ratio had the highest rate of C cell proliferation (Fig. 3B). In a previous investigation, Zheng *et al.* [33] determined that co-culture of human umbilical cord blood-derived MSCs with C cells at a ratio of 2:1 induced more chondrogenic differentiation by adding growth factor FGF-1 into their culture medium. Yang *et al.* [34] reported that a C cells/MSC ratio of 63:1 was required to drive the differentiation of MSCs into a C cell phenotype in a spheroid culture system. The previous studies mentioned above focused on chondrogenic differentiation of MSCs. However, this study is more clinically relevant, as it emphasizes the promotion of the proliferation of mature C cells to overcome the issue of limited resources for clinical purposes. This study also demonstrates that perichondrium is not only an easily accessible resource for P cells that promotes C cell proliferation, but it also promotes unipotent C cell differentiation, as shown in Fig. 3.

The results also showed that P cells rejuvenated and prolonged the lifespan of C cells. As shown in Fig. 3B, the C cells stopped growth and proliferation by Day 75 (P6) in the C2000 group. In contrast, the C cells proliferated up to Day 90 (P8) in the P3000/C2000 group, while they could proliferate up to Day 86 (P9) in the P4000/C2000 group (Fig. 3B and C). Wong *et al.* [35] found that aging cells possess larger cellular sizes, resulting in lower cellular density. Our results demonstrated that the presence of P cells could rejuvenate C cells and prolong their lifespan from P6 to P9 in a dose–dependent manner.

It is well known that C cells undergo senescence easily both *in vitro* and *in vivo*, and this phenomenon is clearly observed in patients with osteoarthritis [36]. A method to expand the lifespan of C cells and maintain their characteristics to prevent de-differentiation was prominent for cartilage regeneration. In our study, C cells were co-cultured with the P cells; as a consequence, the lifespan was extended until P7 in the P1000/C2000 and P2000/C2000 groups, until P8 in the P3000/C2000 group and until P9 (day 86) in the P4000/C2000 group in comparison to P6 (Day 75) in the C2000 group (Fig. 3B and C and Table 1). In the dose–dependent effect of P cells, the total cell number of the C cells increased to 128 times and the Alcian blue^+^ C cells increased to 198 times in the P4000/C2000 group (Table 1). The results demonstrated that P cells dose-dependently promoted the proliferation, rejuvenation, longevity and characteristics of C cells at the latter passages. The Alcian blue^+^ cell numbers shown in Table 1 were estimated by staining area based on hypotheses. Thus, the Alcian blue^+^ cell numbers could not be solid evidence and some analyzes, such as biomarkers of C cells, are recommended to support the positive function of P cells on C cells.

The value of this study is to examine the potential of the P cells on promoting the expansion of C cells, yet maintaining the characteristics of C cells. As the results in Fig. 3C and Table 1, the C cells in the C2000 group could only be passaged up to the sixth generation while in the P4000/C2000 group could be passaged to the ninth generation. Therefore, the maximum cell number in the C2000 group can be expanded to (4.63 ± 0.01) × 10^7^ whereas the P4000/C2000 group can be expanded to (593.1 ± 7.4) × 10^7^. In other words, the ratio of cumulative cell number could increase to 128 times. This achieves the purpose of this study to fulfill clinical needs to provide enough C cells for cartilage regeneration.

To further elucidate whether the factors secreted by P cells to the culture medium have the same effect as the transwell co-culture system on the proliferation of C cells, the conditioned media were prepared with passage criteria dependent/independent on the subconfluency of C cells. As shown in Fig. 4C, crowding of the P cells decreased their own population doubling with dependent passage criteria of C cells, and the P cells’ cumulative population doubling was 2.2 times more in the P4000^§^ group than in the P4000 group. The results indicated that crowding of P cells may secrete more wastes, necrotic/apoptotic factors and toxins, and these deleterious factors inhibit or even reduce the growth of P cells. In contrast, the proliferation of the P cells was promoted with independent passage criteria, indicating markedly more beneficial factors than harmful factors secreted by the P cells in the P4000→C2000^§^ group. This explains why the paracrine effects of the P cells on the proliferation of the C cells were significantly higher in the P4000→C2000^§^ group than in the P4000→C2000 group (Fig. 4B).

Based on the results of the transwell co-culture system and conditioned media system, a large discrepancy in the promotion of C cell proliferation by P cells was still observed between the P4000/C2000 and P4000→C2000^§^ groups (Fig. 4D). As previously mentioned, although more harmful factors might be secreted by crowded P cells in the P4000/C2000 group, its effects on stimulating the proliferation of C cells were still better than those of P cells in the group of P4000→C2000^§^. These results indicated that continuous interaction between P + C cells in the transwell co-culture system provided C cells with the best effect to stimulate proliferation, and the event overcame the deleterious factors arising from the crowded P cells (Fig. 4D). This implies that some fresh factors with activities from the P cells are important for promoting the proliferation of C cells.

Several factors secreted by P cells may explain how they affect the proliferation of C cells with or without cell-to-cell contact. Previous studies have indicated that perichondrocytes secrete factors, such as transforming growth factor β and bone morphogenetic proteins, while C cells produce extracellular matrix proteins, including Type II collagen and aggrecan, during chondrogenic differentiation *in vitro* [37, 38]. Legendre *et al.* [39] demonstrated that a combination of TGF-β1 and BMP-2 enhanced the chondrogenesis of bone marrow-derived stem cells. In Grimaud’s study [40], the TGF-β/BMP superfamily alters the chondrogenic potential of bone marrow-derived mesenchymal progenitor cells and the differentiation and proliferation of articular C cells. Indeed, we have analyzed nascent proteins secreted in the co-cultural medium by LC MS/MS (data not shown). Although not confirmed by further study, fibronectin, apolipoprotein C, TGF-β, IGF-binding protein 2, etc. were found in the co-cultural medium. The expression of dystroglycan has also been reported to influence the structure and function of cells for tissue formation [41].

Whether other factors secreted by P cells also affect the proliferation and chondrogenesis of C cells requires further investigation. As P cells have the characteristics of MSCs and secrete a wide variety of trophic factors, it is possible that these trophic factors are included in some extracellular vesicles (EVs), particularly exosomes, as one of the mediators of MSCs providing paracrine effects for tissue repair. Given that almost all cell types secrete EVs, it is likely that EVs could mediate the effects of the P cells on the C cells. These results may provide a better approach to cartilage regeneration.

From the literature review, most of the studies examined neocartilage formation after subcutaneous implantation in nude mice were carried out at various time points, such as 1, 2, 4, 6, 8 or 12 weeks. The purpose was to understand the progression of neocartilage formation and two time points were used to be chosen because nude mice were susceptible to life threat. Since the aim of our current study was to understand the potentials of the various cells (P, C or P + C cells) on promoting neocartilage formation, the observation at Week 8 post-implantation was optimized for distinguishment. Using a characteristic staining of cartilage, the Alcian blue-positive area and its intensity was the greatest in the implantation group of P + C cells, and was 2.4 times higher than that in the group of C cells (Fig. 5). Hematoxylin and eosin staining also demonstrated typical C cells and cartilage morphology in the implantation group of P + C cells in nude mice. This result indicated that C cells co-cultured with P cells enhanced neocartilage formation. The amount of glycosaminoglycans was significantly higher in the P + C cell group. It seems that the P cells can modulate the proliferation and maintain the characteristics of the C cells, as shown in Fig. 3C.

In the subcutaneous implantation study, results demonstrated that P cells or C cells alone were not able to form an integral cartilage structure, as were P + C cells (Fig. 5). This indicated that P or C cells only may be deficient in certain factors or lack an optimal environment to build a cartilaginous tissue. Consequently, the subcutaneous implantation system allowed us to distinguish different capacities of these cells for cartilaginous tissue formation. Taken together with cell study, P cells possess the characteristic of stem cells, but could not alone form a mature cartilaginous tissue, and neither could C cells alone without the presence of P cells or in an optimal environment, such as in tissue gel. However, in the presence of both P + C cells, a characteristic cartilaginous tissue formed, as shown in Fig. 5. The tissue gel consisting of collagen and hyaluronan may provide a microenvironment temporarily for the neocartilage formation, which was finally degraded. In Fig. 5A, fibrotic tissue formed in F cell-encapsulated tissue gel with filled cells when implanted subcutaneously.

The secretion of glycosaminoglycan is important to the neocartilage, thus, Alcian blue staining at various pH may provide methods to determine the presence of hyaluronan and sulfated glycosaminoglycans, such as chondroitin sulfate. The expression of collagen II, aggrecan, Sox 9 and so on may be also examined to determine chondrogenesis.

In aggregate, this study demonstrated that P cells promoted the proliferation and chondrogenesis of C cells in comparison to individual cultures of P cells or C cells both *in vitro* and *in vivo*. The subcutaneous implantation study significantly demonstrated a synergetic effect of the interaction between P cells and C cells for chondrogenesis and formed an integral cartilage structure. Although the results of Fig. 5 represent an animal study exploring the potential of P cells for further chondrogenesis of C cells, similar results might be obtained in humans, which could offer highly valuable contributions for clinical applications.

Nowadays, the C cell expansion *in vitro* is inevitable in autologous C cell implantation or matrix-induced C cell implantation, thus, the regulation of C cell proliferation and expression will be a potential strategy to improve clinical application. Our current results demonstrated that P cells promoted 128 times of cell expansion more than C cells alone and increased 22% of chondrogenic characteristics to 62% (Table 1), thus, this may provide adequate C cells for clinical application. Besides, the cell tissue gel of collagen and hyaluronan used in our study also provided an optimal formulation to combine with P + C cells for facilitating cartilage regeneration.

*Conflict of interest statement*. None declared.

## Funding 

This work was supported by grants from the Ministry of Science and Technology (MOST 109-2314-B-006-010-MY3), the National Science Council (NSC 93-2314-B-006-044, NSC 96-2314-B-006-047, NSC 97-2314-B-006-046-MY2, NSC 99-2314-B-006-013-MY2, and NSC 99-3111-B-006-002), and the National Cheng Kung University Medical Center (NCKUH-10401004), Tainan, Taiwan.
